# P-1323. Forecasting the incidence of acute respiratory infections in Uzbekistan, 2022-2023

**DOI:** 10.1093/ofid/ofae631.1502

**Published:** 2025-01-29

**Authors:** Rafail Ibragimov, Dilyara Nabirova, Roberta Horth, Botirjon Kurbanov

**Affiliations:** Central Asia Advanced Field Epidemiology Training Program, Tashkent, Toshkent, Uzbekistan; CDC Central Asia office, Almaty, Almaty, Kazakhstan; US Centers for Disease Control and Prevention, Dulles, Virginia; Sanitary-Epidemiological Tranquility and Public Health Committee Tashkent, Uzbekistan, Tashkent, Toshkent, Uzbekistan

## Abstract

**Background:**

Acute respiratory infections (ARIs) are a significant cause of morbidity and mortality worldwide, and this is particularly evident in regions such as Uzbekistan within Central Asia. Forecasting ARIs allows public health authorities to anticipate and prepare for surges in cases. This includes ensuring that healthcare facilities have adequate resources, such as hospital beds, ventilators, medicines, and personal protective equipment (PPE). Therefore, we aimed to predict the incidence of ARIs in Uzbekistan and respond to public health threats in a timely and appropriate manner.

**Figure 1.**
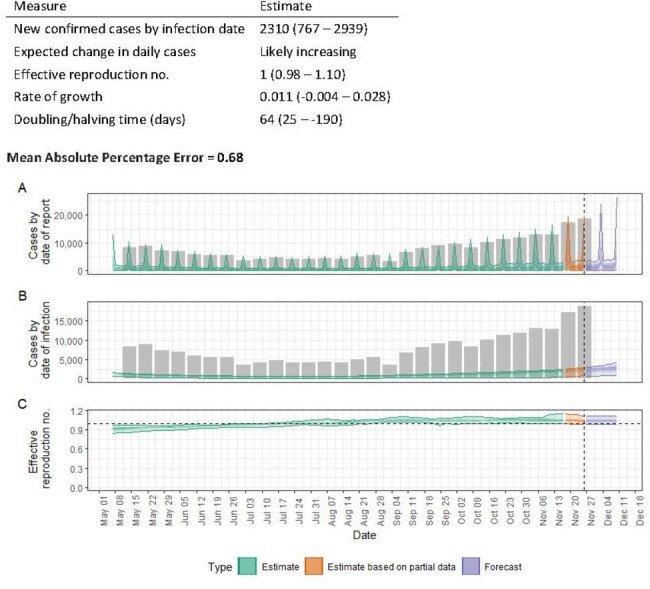
Short-term prediction of incidence acute respiratory infections in Uzbekistan, 2022-2023

**Methods:**

We conducted a retrospective study using weekly data on the incidence of ARIs reported in Uzbekistan between January 2022 and November 2023. Two models were selected to predict the number of ARIs cases. The first model, based on prediction using the EpiNow2 package, was used for short-term prediction for 2 weeks, and the second model, from the nnetar package, based on neural networks for prediction of univariate time series, was used for prediction for the next 12 weeks. Data analysis was performed in R version 4.3.2.

**Figure 2.**
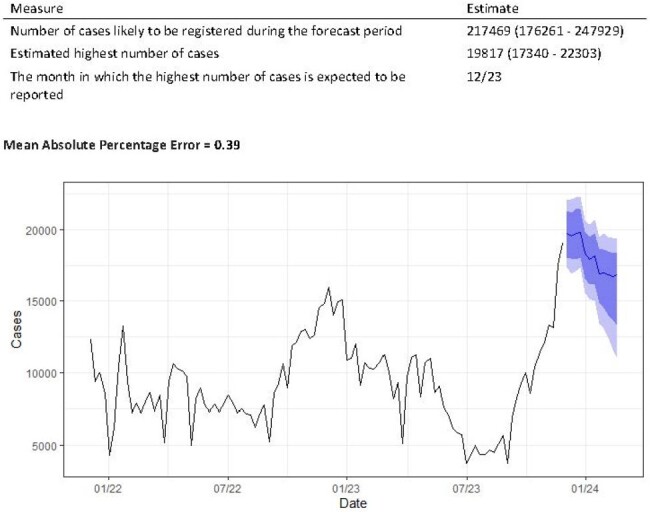
Long term prediction of incidence acute respiratory infections in Uzbekistan, 2022-2023

**Results:**

The short-term forecast for the following 2 weeks showed a possible increase in case numbers (rate of growth = 0.011 (95% CI 0.004 - 0.028)). The model predicts 2,310 (767 - 2,939) new cases by infection date. At the end of the forecast period, the model predicted 5,711 (2,486 - 39,623) new cases of ARIs per week . The long-term model predicted 217,469 (176,261 - 247,929) new infections over the next 12 weeks. At the same time, the peak incidence was predicted to occur in December 2023. In that month, the model predicted 19,817 (17,340 - 22,303) new infections per week. When evaluating the accuracy of the model, we found that the predicted values were lower than the actual number of cases (mean absolute percentage error (MAPE) of the first model = 0.68, MAPE of the second model = 0.39).

**Conclusion:**

The selected models of forecasting make it possible to determine the possible number of cases, although they underestimate values of these cases. The country needs to collect data on risk factors to better predict the incidence of ARIs. However, short- and long-term forecasting models can help to adjust public health response.

**Disclosures:**

**All Authors**: No reported disclosures

